# Influence of Drought and Sowing Time on Protein Composition, Antinutrients, and Mineral Contents of Wheat

**DOI:** 10.1100/2012/485751

**Published:** 2012-05-02

**Authors:** Sondeep Singh, Anil K. Gupta, Narinder Kaur

**Affiliations:** Department of Biochemistry, Punjab Agricultural University, Ludhiana 141004, India

## Abstract

The present study in a two-year experiment investigated the influence of drought and sowing time on protein composition, antinutrients, and mineral contents of wheat whole meal of two genotypes differing in their water requirements. Different thermal conditions prevailing during the grain filling period under different sowing time generated a large effect on the amount of total soluble proteins. Late sown conditions offered higher protein content accompanied by increased albumin-globulin but decreased glutenin content. Fe content was increased to 20–23%; however, tannin decreased to 18–35% under early sown rain-fed conditions as compared to irrigated timely sown conditions in both the genotypes. Activity of trypsin inhibitor was decreased under rain-fed conditions in both genotypes. This study inferred that variable sowing times and irrigation practices can be used for inducing variation in different wheat whole meal quality characteristics. Lower temperature prevailing under early sown rain-fed conditions; resulted in higher protein content. Higher Fe and lower tannin contents were reported under early sown rain-fed conditions however, late sown conditions offered an increase in phytic acid accompanied by decreased micronutrients and glutenin contents.

## 1. Introduction

Wheat (*Triticum aestivum *L.), the most important cereal crop along with rice and corn, feeds most of the world's population. It is contributing to 28% of the world edible dry matter and up to 60% of the daily calorie intake in several developing countries. Wheat consumption is increasing worldwide as a result of higher income levels, urbanization, and substitution with other cereals. Therefore, the nutritional quality of the wheat whole meal has a significant impact on human health and well-being especially in the developing world [1].

Wheat endosperm acts as a starch storage region of the kernel while germ is a concentrated source of minerals and protein. Globally, wheat is the leading source of vegetable protein in human food having higher protein content than the other major cereals. Amount as well as composition of grain proteins determines the protein quality and hence end-use of wheat. The gluten proteins, the gliadins, and glutenins constitute up to 80–85% of total flour protein, and they confer properties of elasticity and extensibility that are essential for functionality of wheat whole meals [2]. Apart from the gluten proteins, albumins and globulins constituting 10–22% of total flour protein [3] are important from nutritional point because of high amounts of essential amino acids.

Mineral nutrients play a fundamental role in the biochemical and physiological functions of biological systems. Micronutrient malnutrition particularly of Fe and Zn is a growing concern worldwide. It is mainly due to extensive consumption of staple cereals which have low amount and availability of micronutrients [4]. Modern wheat (*T*.* aestivum*) cultivars are poor in Fe and Zn contents as compared to wild and primitive wheat such as einkorn wheat (*Triticum monococum*), emmer wheat (*Triticum dicocum*), and wild emmer wheat (*Triticum dicoccoides*) [5]. One sustainable agricultural approach to reducing micronutrient malnutrition globally is to enrich staple food crops with micronutrients or decreasing antinutrient substances that inhibit micronutrient bioavailability. So the nutritional quality of wheat whole meal is further dependent on the status of antinutrients such as phytates, tannins, and protease inhibitors in grains.

Phytate or phytic acid accounts for as much as 85% of the total phosphorus content of cereal grains and significantly influences the functional and nutritional properties of foods. Due to its strong binding capacity, phytic acid readily forms insoluble complexes with multivalent cations and proteins at physiological pH and hence renders several minerals biologically unavailable to animals and humans [6]. Low phytic acid-containing cereals, therefore, are desirable from nutritional point. Tannins are polyhydric phenols which form insoluble complexes with proteins, carbohydrates, and lipids leading to reduction in digestibility of these nutrients. Other effects that have been attributed to tannins include damage to the intestinal tract and interference with the absorption of iron and a possible carcinogenic effect [7]. Similarly protease inhibitors are known to cause growth inhibition by interfering with digestion causing pancreatic hypertrophy and metabolic disturbance of sulfur amino acid utilization [8].

During the past several decades, the primary objective of plant breeding programs has been to increase yield, a quest that will remain a principal concern in providing the calorie intake required for the growing world population. However, equally important but largely overlooked in breeding programs is the nutritional quality of wheat whole meal particularly the protein quality and micronutrients concentrations and their bioavailability [5].

The wheat quality characteristics are usually influenced by genotype, environmental factors, and interactions between genotype and environment. Adverse environmental conditions such as extreme temperature and drought during the anthesis and grain filling period have been identified as major constraints to wheat protein content and composition [9, 10]. Most of the earlier studies were focused on the effect of either heat or drought stress during grain filling on composition of proteins and starch characteristics. Few studies have examined the combined effect of high temperature and drought on protein and gluten contents with limited emphasis on nutritional quality. However, little is known concerning the combined effect of sowing time and drought stress on nutritional quality of wheat whole meal. In our earlier work we analysed the effect of rain-fed and different sowing times on monomeric/polymeric proteins and starch pasting characteristics of wheat whole meal [11]. The study clearly indicated the significant variation in the content and composition of storage proteins under different sowing conditions. Rain-fed conditions resulted in generation of new correlations among various protein fractions and starch pasting characteristics, which were far from those observed under irrigated conditions. The study suggested that utilization of these new correlation trends observed between different protein components under rain-fed conditions could be useful for enhancing protein content without affecting protein quality of wheat grains. However, the work was confined up to the level of monomeric and polymeric protein fractions and starch pasting characteristics. In the present study, therefore, we are attempting to further fractionate the protein to albumin-globulins and gliadin components and to observe the variation in the minerals and antinutrients, to better understand the effect of varying sowing conditions on wheat whole-meal quality.

## 2. Materials and Methods

### 2.1. Plant Material and Experimental Conditions

Two wheat (*T*.* aestivum *L.) genotypes, PBW 343 (high yielding, drought susceptible) and C 306 (drought tolerant), were grown in fields of the Punjab Agricultural University, Ludhiana, (30°54′N, 75°48′E, elevation 247 m above mean sea level), India. The experimental soil was loamy sand with pH about 7.8–8.0. The seeds were sown in 2 m^2^ plots with a row space of 20 cm. The sowing dates were early sown (ES) (15 October), timely sown (TS) (15 November), and late sown (LS) (15 December) in 2007 (year I) and 2008 (year II). The experimental design was a randomized complete block with three replications. Under the irrigated treatment, plants were watered throughout the period from sowing to maturity according to the recommended agronomic practices [12]. All irrigations were withheld from the plants subjected to the drought treatment except the presowing irrigation for field preparation. Therefore, the drought-treated plants received water only available through rainfall. The weather data of total rainfall and evapotranspiration rate for the crop season in both years was collected from the field meteorological observatory. Total rainfall as an average of two years received by the crop from sowing to maturity was 94, 81, and 82 mm, while during grain filling period was 23, 55, and 45 mm for early, timely and late sown crops, respectively. During the crop season rainfall was scanty while evapotranspiration was higher, which helped in the development of severe drought stress.

### 2.2. Grain Plumpness

Wheat grains were separated according to the grain diameters (>2.8 mm and <2.5 mm) using grain sorter (Sortimat, Germany) fitted with respective sized meshes. The grains of different diameters were collected and then weighed on an electronic balance and expressed in percentage.

### 2.3. Test Weight

 Test weight was determined using the apparatus developed by the Directorate of Wheat Research, Karnal, India, which employs a standard container of 100 mL capacity. The container is filled with the sample of wheat grains by removing all shrunken and broken kernels and other foreign material. The grains were weighed and the test weight expressed in kg/hectoliter (hl) [13].

### 2.4. Milling and Protein Fractionation

Grains from each treatment were finally milled using Cyclotec 1093 sample mill (Foss, Tecator, Sweden) to obtain wheat whole meal in both the years. Protein fractionation was carried out from wheat whole meal according to Triboi et al. [10] with a little modification. Briefly, the protein fractions albumin-globulin, amphiphil, gliadin, and glutenin were sequentially extracted from 800 mg of flour. During each extraction step, the samples were continuously shaken in a volumetric flask (100 mL) placed in a temperature controlled shaker (orbital shaking incubator CIS-24, Remi, India) for 60 min. Soluble and insoluble fractions were separated by centrifugation at 8,000 × g (Sigma, USA) for 30 min at the extraction temperature. Albumins-globulins were extracted at 4°C with 25 mL 0.05 M NaCl, 0.05 M sodium phosphate buffer pH 7.8. After removal of amphiphilic proteins by extraction at 4°C from the previous pellet with 25 mL 2% (v/v) Triton X-114, 0.1 M NaCl, 0.05 M sodium phosphate buffer pH 7.8, gliadins were extracted at 20°C from the previous pellet with 25 mL 70% (v/v) ethanol. Glutenins were extracted at 20°C from the previous pellet with 25 mL 2% (w/v) SDS (sodium dodecyl sulfate), 2% (v/v) *β*-mercaptoethanol, 0.05 M tetraborate buffer pH 8.5. After centrifugation, the glutenins were recovered in the supernatant.

### 2.5. Antinutrients

Antinutrients like phytic acid was extracted from wheat whole meal with 1.2% HCl and precipitated with 0.4% FeCl_3_, and inorganic phosphorus was estimated as described previously [14]. Trypsin inhibitor was isolated and quantified by inhibiting the bovine trypsin by using N-*α*-benzoyl-DL-arginine-*p*-nitroanilide as a substrate [15]. One trypsin inhibitor unit is defined as the quantity of inhibitor which inhibits 50% of trypsin inhibitor activity. Tannins were extracted and estimated as described earlier [16]. Briefly, tannins were extracted twice from wheat whole meal with boiling water. All the fractions were filtered through the Whatman no. 1 filter paper and pooled. The intensity of blue colour developed after addition of Folin-Denis' reagent and sodium carbonate solutions was measured at 700 nm.

### 2.6. Minerals

Wheat whole meal was analysed for Fe and Zn contents using atomic absorption spectrophotometer (AAS 240, Varian). Flour samples were digested in a mixture of nitric acid:perchloric acid (10 : 1 v/v); volume was made with quartz glass distilled water and analysed by AAS 240. 

### 2.7. Statistical Analysis

The results are expressed as mean ± standard deviation of three replicates obtained from three different sowing plots in the fields. A completely randomized design was used for analysis of variance (ANOVA) using CPCS 1 software package. Correlation analysis was carried out using MS Excel 2003. Statistical significance was set at *P* ≤ 0.05, except where specified.

## 3. Results

**Figure 1:**
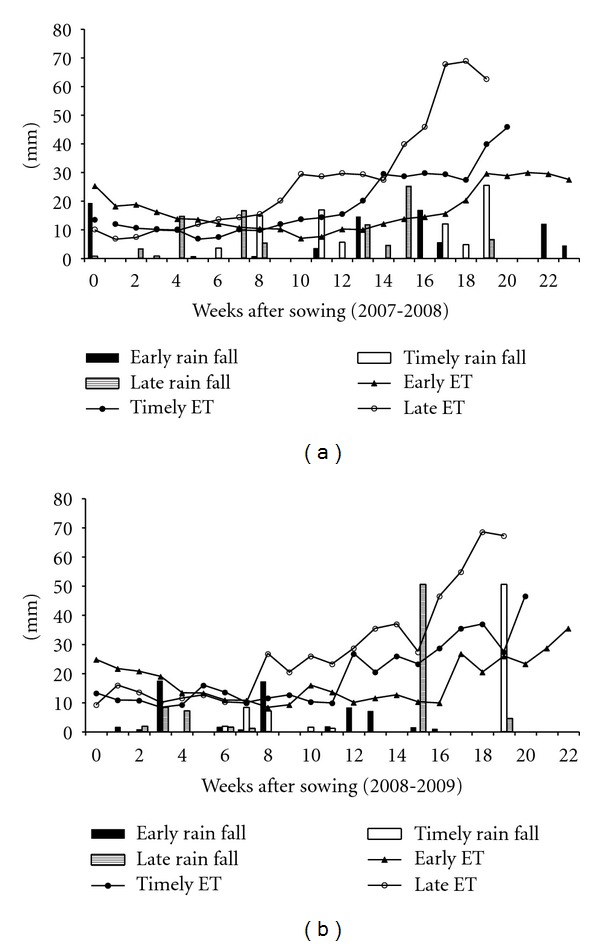
Patterns of total rain fall and evapotranspiration during the wheat growing season for the year 2007-2008 (year I) and 2008-2009 (year II). ET, evapotranspiration.

**Figure 2:**
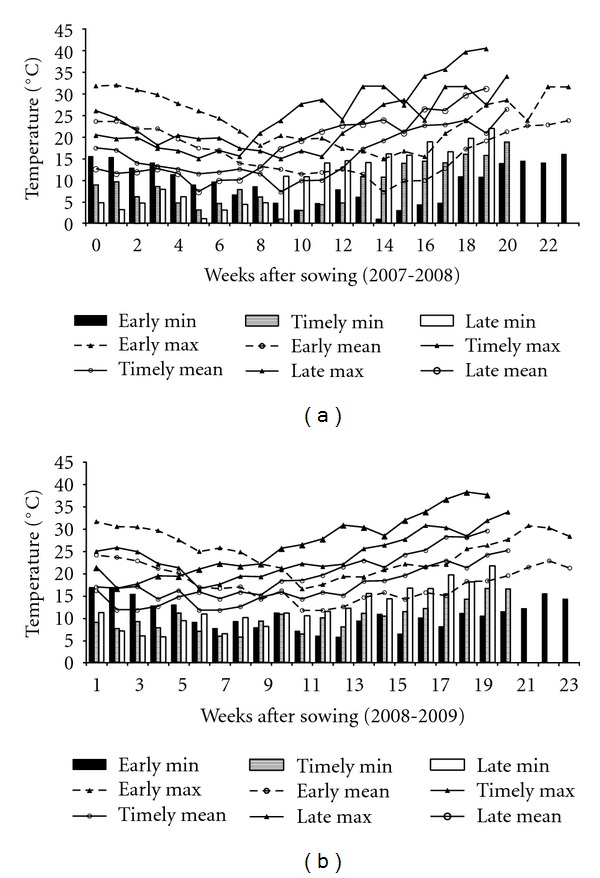
Patterns of weekly temperatures during the wheat growing season for the year 2007-2008 (year I) and 2008-2009 (year II).

The different sowing times selected in this study resulted in exposure of plants to varied temperatures (Figure 1) and rainfall (Figure 2) events before and during grain filling periods. The mean maximum temperatures during grain filling periods under early sown (ES), timely sown (TS), and late sown (LS) conditions as an average of both the years were 22.4°C, 26.3°C, and 29.4°C, respectively. Similarly, mean minimum temperatures were 8.8°C, 11.8°C, and 14.2°C in ES, TS, and LS conditions, respectively. Diverse temperature range and amount of rainfall in different sowing times resulted in significant differences in test weight and grain plumpness (Table 1) for both genotypes.

### 3.1. Flour Protein Fractions

Amount of total soluble protein (TSP) was higher in genotype C 306 as compared to PBW 343 under all treatments (Table 2). Rain-fed (RF) conditions resulted in decrease in TSP under timely sown (TS) and late sown (LS) crop whereas significant increase was observed under early sown (ES) conditions. Crop in irrigated ES conditions accumulated lesser TSP; however, a significant increase in TSP accumulation was observed for LS conditions in both genotypes. This resulted in higher grain protein percentage due to delayed sowing (Table 2). 

Different protein fractions were also influenced by sowing times and RF conditions in both genotypes (Table 2). Albumin-globulin and gliadin proteins ranged from 9 to 13% and 21 to 32% of total protein, respectively, under irrigated conditions (Table 2). When compared to irrigated TS conditions, albumin-globulin proteins were enhanced under irrigated LS as well as under RF conditions in both genotypes. Both in irrigated + TS and RF + TS conditions, the gliadin content was significantly low in C 306 as compared to PBW 343 (Table 2). However, under RF + ES conditions, relative content of gliadin decreased in both genotypes as compared to RF + TS conditions. This led to lower gliadin percentage in C 306 as compared to PBW 343 under RF + ES conditions (Table 2). In PBW 343, LS conditions resulted in lower accumulation of gliadin both under irrigated and rain-fed conditions, and such an effect was also observed in crop of C 306. Rain-fed conditions in general resulted in lowering of glutenin in ES and TS crop. Grains of C 306 obtained from ES and TS crops had relatively higher percentage of glutenin as compared to the respective grains of PBW 343 (Table 2). However, this trend is reversed in LS crop (Table 2).

### 3.2. Mineral Contents

In the present study, different sowing times and irrigation/RF conditions resulted in significant variation in grain micronutrients particularly in Fe contents. In general, grain Fe and Zn contents ranged from 26 to 41 and 19 to 24 mg/Kg, respectively, under irrigated conditions (Table 3). Fe content was significantly higher in PBW 343 as compared to C 306 under irrigated ES and TS conditions as well as under RF conditions. However, under irrigated LS conditions, C 306 registered higher Fe content (Table 3). Effect of sowing time on Fe content was variable among the genotypes and across the years. On comparing the average of both years, 22% increase in Fe content was recorded under irrigated ES conditions in PBW 343 as compared to irrigated TS conditions. Under RF + ES conditions, both genotypes registered 5–7% increase in Fe content as compared to RF + TS conditions, and 20–23% increase as compared to irrigated TS conditions (Table 3). In contrast, LS+RF conditions resulted in 20–24% decrease in Fe content in both the genotypes as compared to TS+RF conditions. Further, an increase (5–21%) in Fe content was observed in both the genotypes in response to RF conditions except in C 306 under LS conditions. Effect of sowing time and RF conditions on Zn contents was not significant in both years. In general, Zn content increased in response to RF conditions under ES conditions but decreased under TS and LS conditions in both genotypes (Table 3). 

 Cultivar C 306 had lower tannin content as compared to PBW 343 under irrigated as well as RF conditions (Table 4). Trypsin inhibitor activity was more in C 306 as compared to PBW 343 irrespective of sowing time and irrigation conditions. The ES crop appeared to have higher trypsin inhibitor activity (Table 4). In ES and TS crops, on an average PBW 343 had slightly higher phytic acid content in irrigated crop (Table 4).

## 4. Discussion

Proteins are the most important components of wheat grains governing end-use quality. Both amount and composition of proteins determine the protein quality and hence end-use quality of wheat. Environmental conditions during grain filling influence the accumulation of protein in the developing wheat kernel and can alter the functional properties of the resulting flour. Variations in both protein content and composition significantly modify flour quality for different end products. Although grain protein composition depends primarily on genotype, it is significantly affected by environmental factors and their interactions [17]. Increase in flour protein under water deficit conditions has been reported mainly due to higher rates of accumulation of grain nitrogen and lower rates of accumulation of carbohydrates. Irrigation, on the other hand, may decrease flour protein content by dilution of nitrogen with carbohydrates [18]. Changing the sowing time generated a large effect on the amount of TSP (Table 2), probably driven by the different thermal conditions prevailing during the grain filling period (Figure 1). This was particularly evident on comparing the early and late sowings. Similar results have been reported earlier [19]. Although albumin-globulins do not have significant effect on dough quality, but they are important from nutritional point of view due to high content of essential amino acids in them. Hurkman et al. have reported accumulation of albumin-globulins in response to high temperature in wheat grains [20].

It appears that optimum temperature of gliadin synthesis in grains is genotype dependent. The differential effect of temperature leading to relatively higher production of gliadins resulting in reduced dough strength has been reported earlier [19]. The results indicate that C 306 and PBW 343 have different optima temperature for synthesis of glutenin. An increased grain protein and gluten content in response to late water stress as compared to the fully irrigated treatment in a winter bread variety has also been reported earlier [21]. Labuschagne et al. also reported different temperature requirements of polymeric glutenin accumulation in different wheat cultivars [22]. LS conditions are offering higher protein content accompanied by increased albumin-globulin; however, decrease in glutenin content made the flour technically of poor quality. After anthesis, environmental conditions primarily affect kernel size, protein concentration and composition. Temperature and rain fall during grain filling strongly affect grain protein content and gliadin to HMW-GS and LMW-GS ratios [23]. An evaluation of drought and heat effects on wheat in a Mediterranean climate showed the highest grain protein content under warm dry rain-fed conditions and the lowest in the irrigated environment [24].

The gluten proteins, glutenin and gliadin, are responsible for flexibility and extensibility of dough. The deterioration in dough quality has been attributed to decline in glutenin-to-gliadin ratio. The glutenin-to-gliadin ratio was higher in drought-tolerant genotype C 306 as compared to PBW 343 when crop was raised under ES and TS conditions. Under rain-fed conditions, this ratio increased in C 306 whereas it either remained unchanged or declined in PBW 343 on the basis of data of two years. Therefore, it can be concluded that dough quality in C 306 flour is not adversely affected when crop is grown under rain-fed conditions as ES and TS crops. However, when crop is exposed to both drought and high temperature (Figure 1) during LS conditions, the glutenin-to-gliadin ratio declines both in C 306 and PBW 343 (Table 2).

Changes in the amount of gluten transcripts or in the temporal regulation of gluten protein genes in response to environmental conditions could lead to alterations in flour quality. Ratios of the different classes of proteins or of specific proteins within each class could change and thus affect the formation of glutenin polymers [25]. Water deficit and temperature being the important environmental conditions influencing the amount, composition, and/or polymerization of the wheat storage proteins have been reported in earlier studies [21, 26].

Among grain mineral nutrients, Zn and Fe deficiencies are the most important global challenge. According to the World Health Organization, deficiencies in Zn and Fe rank 5th and 6th, respectively, among the risk factors responsible for illnesses in developing countries. Deficiency in Zn and Fe afflicts over three billion people worldwide resulting in overall poor health, anemia, increased mortality rates, and lower worker productivity [1]. Producing micronutrient-enriched cereals and improving their bioavailability are considered promising and cost-effective approaches for diminishing malnutrition.

Gomez-Becerra et al. reported that the concentration of grain's Fe and Zn in modern wheat cultivars is on an average around 35 and 25 mg/Kg, respectively [27]. Peleg et al. have reported that water stress conditions either result in increase, decrease, or no changes in grain micronutrients in wheat [1]. In other words, no definite trend on accumulation of micronutrient under water deficit conditions can be predicted. In the present work, just by changing sowing time and irrigation conditions, a significant variation in micronutrient particularly grain Fe contents (Table 3) and protein composition was observed. This variation could be useful for selection of superior germplasm for breeding programs aimed at mineral biofortification. For example, under RF conditions, an increase in grain Fe content in both genotypes in a two-year trial were observed (Table 3). Obviously these data gave an encouraging indication for a study involving large number of wheat genotypes under restricted/controlled irrigation to increase grain Fe content. Increasing evidence suggests that enhancement of grain protein content of wheat could also greatly contribute to biofortification with micronutrients [28–30]. Distelfeld et al. suggested the possible role of proteins as potential candidates for chelating micronutrients [31]. Further, cereal grains are inherently poor in concentration of micronutrients and rich in compounds depressing their bioavailability such as phytic acid [32]. At physiological pH, phytic acid due to high negative charge density acts as a strong chelator of positively charged mineral cations such as Fe and Zn [33]. When comparing the average data of phytic acid contents for two years (Table 4), minimum phytic acid content was observed in grains of ES crop. Under irrigated conditions, PBW 343 has a higher phytic acid content in ES and TS crops. Maximum content of phytic acid was observed in LS crop of PBW 343. Although there are reports that Zn and Fe content increased with higher phytic acid and protein content [34], such a behavior was not observed with respect to phytic acid in the same genotype when sown under different climatic conditions. In LS crop, combination of higher temperature and water-deficit conditions probably caused maximum phytic acid content in the grains.

Both the genotypes registered 18–35% reduction in tannin content under ES+RF conditions when compared to TS-irrigated conditions. The inhibitory effects on absorption and utilization of minerals such as iron and zinc have also been attributed to the presence of trypsin inhibitors [33]. Effect of ES conditions was variable among the genotypes since inhibitor activity increased in C 306 but decreased in PBW 343 when compared to irrigated TS conditions (Table 4). However, RF conditions clearly resulted in significant decrease in trypsin inhibitor as well as tannin contents in both genotypes. This suggested that ES+RF conditions resulted not only in higher grain Fe content (Table 3) but also lower antinutrients (Table 4) associated with higher grain protein contents (Table 2). In contrast, in the present study LS conditions are offering an increase in phytic acid content accompanied by decreased micronutrients (Table 3) and glutenin content (Table 2), thereby decreasing the grain nutritional quality. So lower temperature of ES conditions (around 22°C during grain filling in present study) along with limited irrigation could offer a new approach for biofortification of wheat grains without any additional economic inputs.

## Figures and Tables

**Table 1:** Effect of sowing time on test weight and grain plumpness of drought-susceptible (PBW 343) and drought-tolerant (C 306) wheat genotypes grown under irrigated and rain-fed conditions. Values are mean ± standard deviation of three plots; CD, critical difference; hl, hectoliter; NS, not significant.

Year	Sowing time	Genotype	Test weight (Kg/hl)		Grain plumpness > 2.8 mm(%)		Grain plumpness < 2.5 mm (%)	
			Treatment		Treatment		Treatment	
			Irrigated	Rain fed	Irrigated	Rain fed	Irrigated	Rain fed
Year I (2008)	Early sown	PBW 343	71.82 ± 0.40	66.50 ± 3.98	26.71 ± 1.97	39.12 ± 0.75	21.21 ± 0.59	17.34 ± 0.27
		C 306	77.81 ± 0.96	71.48 ± 0.51	53.73 ± 2.70	23.48 ± 1.25	12.16 ± 0.54	29.24 ± 1.23
	Timely sown	PBW 343	72.74 ± 0.04	68.89 ± 0.45	24.70 ± 0.36	7.79 ± 1.87	20.25 ± 0.71	40.32 ± 1.95
		C 306	75.17 ± 2.28	76.94 ± 0.30	56.18 ± 6.40	41.65 ± 1.00	13.22 ± 5.29	19.26 ± 0.28
	Late sown	PBW 343	70.18 ± 0.14	71.78 ± 0.08	9.16 ± 0.81	14.52 ± 0.54	38.61 ± 1.44	31.74 ± 1.59
		C 306	76.87 ± 0.64	74.04 ± 0.09	63.17 ± 1.12	39.94 ± 0.06	9.83 ± 0.85	19.42 ± 0.15

Year II (2009)	Early sown	PBW 343	74.08 ± 0.60	72.04 ± 0.14	66.87 ± 17.77	36.42 ± 0.53	7.02 ± 4.21	21.85 ± 0.57
		C 306	78.95 ± 1.33	77.70 ± 0.87	65.55 ± 3.00	42.69 ± 8.24	8.54 ± 0.77	18.63 ± 3.10
	Timely sown	PBW 343	75.19 ± 1.40	72.45 ± 1.21	58.82 ± 16.80	33.40 ± 3.59	7.82 ± 4.90	22.56 ± 2.02
		C 306	78.17 ± 2.28	77.26 ± 0.45	63.84 ± 10.43	43.25 ± 7.60	9.22 ± 4.09	19.39 ± 2.97
	Late sown	PBW 343	68.09 ± 2.23	72.66 ± 1.02	13.49 ± 5.52	13.95 ± 0.81	32.42 ± 6.81	34.03 ± 0.96
		C 306	76.86 ± 1.15	75.90 ± 1.67	37.00 ± 6.78	31.84 ± 7.18	20.09 ± 3.28	24.53 ± 2.55

			Test weight		Grain plumpness > 2.8 mm		Grain plumpness < 2.5 mm	
			Year I	Year II	Year I	Year II	Year I	Year II

CD *P* ≤ 0.05	Sowing time		1.12	1.10	1.55	3.09	1.55	3.09
	Genotype		0.91	0.90	1.27	2.52	1.27	2.52
	Treatment		0.91	NS	1.27	2.52	1.27	2.52

**Table 2:** Effect of sowing time on albumin-globulin, gliadin, and glutenin proteins in mature grains of drought-susceptible (PBW 343) and drought-tolerant (C 306) wheat genotypes grown under irrigated and rain-fed conditions. Values are mean ± SD of three replicates; TSP, total soluble proteins; NS, not significant.

Year	Sowing time	Genotype	TSP (%)		Amphiphil (% of total protein)		Albumin-globulin (% of total protein)		Gliadin (% of total protein)		Glutenin (% of total protein	
			Treatment		Treatment		Treatment		Treatment		Treatment	
			Irrigated	Rain fed	Irrigated	Rain fed	Irrigated	Rain fed	Irrigated	Rain fed	Irrigated	Rain fed

	Early sown	PBW 343	9.32 ± 0.79	11.62 ± 0.59	5.06 ± 0.49	6.13 ± 0.10	11.42 ± 0.41	13.46 ± 0.15	31.62 ± 1.08	27.54 ± 0.65	39.31 ± 1.36	34.36 ± 0.74
		C 306	10.17 ± 0.75	14.02 ± 0.61	3.43 ± 0.31	6.74 ± 0.12	12.25 ± 0.25	13.90 ± 0.12	30.40 ± 0.55	26.12 ± 0.94	44.25 ± 1.34	40.18 ± 0.66
Year I (2008)	Timely sown	PBW 343	10.51 ± 0.13	9.40 ± 0.51	4.60 ± 0.39	5.75 ± 0.33	10.79 ± 0.21	13.97 ± 0.50	31.26 ± 0.70	36.56 ± 0.95	44.56 ± 1.46	41.73 ± 0.97
		C 306	12.48 ± 0.21	11.62 ± 0.29	5.37 ± 0.10	7.12 ± 0.19	11.68 ± 0.34	13.53 ± 0.29	20.59 ± 1.50	17.87 ± 0.69	48.37 ± 2.14	44.37 ± 1.06
	Late sown	PBW 343	12.31 ± 0.17	11.60 ± 0.49	5.76 ± 0.18	6.28 ± 0.17	13.01 ± 0.29	13.46 ± 0.31	22.33 ± 0.56	27.14 ± 0.38	46.69 ± 1.24	44.08 ± 1.06
		C 306	13.76 ± 0.59	11.97 ± 0.24	5.25 ± 0.12	7.27 ± 0.16	11.94 ± 0.16	13.35 ± 0.35	29.95 ± 1.29	34.42 ± 0.51	40.91 ± 0.56	41.93 ± 0.88

	Early sown	PBW 343	10.09 ± 0.45	12.22 ± 0.15	6.16 ± 0.19	6.85 ± 0.17	9.17 ± 0.51	10.42 ± 0.21	29.51 ± 0.88	26.89 ± 0.45	41.08 ± 1.32	35.07 ± 0.96
		C 306	10.26 ± 0.51	12.74 ± 0.14	4.15 ± 0.21	5.91 ± 0.12	10.12 ± 0.24	11.30 ± 0.22	28.05 ± 0.68	23.25 ± 0.46	45.47 ± 1.80	41.44 ± 1.36
Year II (2009)	Timely sown	PBW 343	10.94 ± 0.81	10.17 ± 0.56	3.17 ± 0.10	4.83 ± 0.19	9.17 ± 0.15	11.67 ± 0.24	28.26 ± 0.55	35.26 ± 0.69	47.47 ± 0.88	40.11 ± 1.73
		C 306	12.82 ± 0.16	10.60 ± 0.17	4.60 ± 0.23	6.15 ± 0.18	11.15 ± 0.19	12.86 ± 0.37	24.40 ± 0.56	19.13 ± 0.33	51.09 ± 1.58	43.52 ± 0.72
	Late sown	PBW 343	11.54 ± 0.13	10.43 ± 0.34	5.81 ± 0.11	6.90 ± 0.15	12.44 ± 0.33	12.80 ± 0.65	23.67 ± 0.50	30.14 ± 0.70	48.55 ± 1.57	45.96 ± 2.23
		C 306	13.16 ± 0.77	11.03 ± 0.82	5.14 ± 0.14	8.84 ± 0.18	10.30 ± 0.35	11.62 ± 0.29	27.45 ± 0.48	30.12 ± 0.64	42.24 ± 1.04	44.21 ± 1.10

			TSP		Amphiphil		Albumin-globulin		Gliadin		Glutenin	
			Year I	Year II	Year I	Year II	Year I	Year II	Year I	Year II	Year I	Year II

CD *P* ≤ 0.05	Sowing time		0.15	0.19	0.36	0.24	NS	0.48	1.14	NS	1.68	2.00
	Genotype		0.12	0.15	NS	NS	NS	NS	0.93	1.19	1.37	NS
	Treatment		0.12	0.15	0.29	0.19	0.34	0.39	NS	NS	1.37	1.63

**Table 3:** Effect of sowing time on iron (mg/Kg) and zinc (mg/Kg) contents in mature grains of drought-susceptible (PBW 343) and drought-tolerant (C 306) wheat genotypes grown under irrigated and rain-fed conditions. Values are mean ± standard deviation of three replicates; CD, critical difference; NS, not significant.

Year	Sowing Time	Genotype	Iron (mg/Kg)		Zinc (mg/Kg)	
			Treatment		Treatment	
			Irrigated	Rain fed	Irrigated	Rain fed
Year I (2008)	Early sown	PBW 343	40.85 ± 0.58	43.85 ± 0.43	23.06 ± 0.27	23.42 ± 0.07
		C 306	35.50 ± 0.98	42.95 ± 1.78	19.28 ± 2.52	23.04 ± 0.60
	Timely sown	PBW 343	27.35 ± 0.37	40.05 ± 0.18	22.36 ± 0.42	18.14 ± 3.88
		C 306	26.35 ± 0.54	37.15 ± 1.44	22.61 ± 0.25	22.49 ± 0.32
	Late sown	PBW 343	33.55 ± 1.36	35.70 ± 0.73	22.53 ± 0.34	18.10 ± 3.04
		C 306	35.05 ± 1.32	30.20 ± 1.31	19.79 ± 2.14	18.16 ± 5.45

Year II (2009)	Early sown	PBW 343	37.80 ± 1.75	42.05 ± 1.87	22.82 ± 0.12	23.89 ± 0.10
		C 306	26.35 ± 1.60	34.20 ± 2.00	22.57 ± 0.18	23.64 ± 0.20
	Timely sown	PBW 343	36.95 ± 2.07	41.70 ± 0.67	23.01 ± 0.48	23.67 ± 0.18
		C 306	36.25 ± 0.80	34.80 ± 2.02	23.48 ± 0.19	22.30 ± 0.26
	Late sown	PBW 343	29.10 ± 0.87	30.15 ± 1.33	23.56 ± 0.14	23.26 ± 0.09
		C 306	31.20 ± 2.78	29.32 ± 3.63	23.70 ± 0.14	23.00 ± 0.20

			Iron		Zinc	
			Year I	Year II	Year I	Year II

CD *P* ≤ 0.05	Sowing time		1.24	2.34	NS	NS
	Genotype		1.01	1.91	NS	0.21
	Treatment		1.01	1.91	NS	NS

**Table 4:** Effect of sowing time on tannin (*μ*g g^−1^ DW), phytic acid (mg g^−1^ DW), and trypsin inhibitor (units g^−1^ DW) contents in mature grains of drought-susceptible (PBW 343) and drought tolerant (C 306) wheat genotypes grown under irrigated and rain-fed conditions. Values are mean ± standard deviation of three replicates; CD, critical difference; NS, not significant; One inhibitor unit is defined as the quantity of inhibitor which inhibits 50% of trypsin activity.

Year	Sowing time	Genotype	Tannin (*μ*g g^−1^ DW)		Phytic acid (mg g^−1^ DW)		Trypsin inhibitors (units g^−1^ DW)	
			Treatment		Treatment		Treatment	
			Irrigated	Rain fed	Irrigated	Rain fed	Irrigated	Rain fed
Year I (2008)	Early sown	PBW 343	1608 ± 63.68	1382 ± 50.69	8.49 ± 0.50	6.00 ± 0.42	31.94 ± 0.98	33.80 ± 2.27
		C 306	1083 ± 48.03	1010 ± 18.03	7.85 ± 0.70	8.84 ± 0.68	45.83 ± 1.96	42.13 ± 1.13
	Timely sown	PBW 343	1840 ± 24.74	1689 ± 29.68	7.95 ± 0.62	9.38 ± 0.96	31.48 ± 3.97	27.31 ± 2.84
		C 306	1434 ± 16.00	1353 ± 33.79	8.00 ± 0.53	9.48 ± 1.02	40.28 ± 5.89	27.59 ± 6.57
	Late sown	PBW 343	1864 ± 20.87	1842 ± 19.00	9.58 ± 0.82	7.51 ± 0.45	29.63 ± 2.84	21.76 ± 2.27
		C 306	1767 ± 10.87	1470 ± 30.69	8.84 ± 0.40	6.62 ± 0.84	36.11 ± 5.97	32.87 ± 1.13

Year II (2009)	Early sown	PBW 343	1901 ± 24.17	1783 ± 32.50	7.70 ± 1.12	9.33 ± 1.28	34.26 ± 1.40	31.48 ± 1.57
		C 306	1426 ± 25.50	913 ± 12.50	7.56 ± 0.97	7.48 ± 0.64	46.30 ± 1.60	45.37 ± 1.52
	Timely sown	PBW 343	2020 ± 19.14	1426 ± 14.00	8.52 ± 0.36	9.26 ± 0.23	39.81 ± 1.13	31.02 ± 1.23
		C 306	1545 ± 29.48	1307 ± 19.67	9.74 ± 1.11	7.33 ± 0.59	41.20 ± 2.84	30.56 ± 0.98
	Late sown	PBW 343	1901 ± 22.03	1664 ± 27.57	10.00 ± 0.44	13.93 ± 0.28	30.56 ± 3.54	24.54 ± 1.50
		C 306	1248 ± 26.50	832 ± 20.88	9.22 ± 0.56	10.59 ± 0.90	39.81 ± 3.97	32.41 ± 1.45

			Tannin		Phytic acid		Trypsin inhibitors	
			Year I	Year II	Year I	Year II	Year I	Year II

CD *P* ≤ 0.05	Sowing time		25.56	17.27	0.58	0.66	3.77	2.5
	Genotype		20.87	14.10	NS	0.54	3.08	2.05
	Treatment		20.87	14.10	0.48	0.54	3.08	2.05
